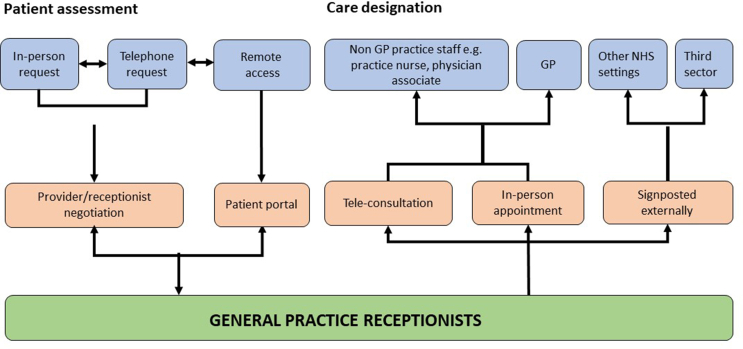# “*You're only a receptionist, what do you want to know for?*”: Street-level bureaucracy on the front line of primary care in the United Kingdom

**DOI:** 10.1016/j.heliyon.2023.e21298

**Published:** 2023-11-13

**Authors:** Ian Litchfield, Nicola Gale, Michael Burrows, Sheila Greenfield

**Affiliations:** a Institute of Applied Health Research, College of Medical and Dental Sciences, University of Birmingham, Birmingham, UK; b Health Services Management Centre, School of Social Policy, University of Birmingham, UK; c Department of Forensic Psychology, School for Health and Life Sciences, Coventry University, UK

**Keywords:** Street-level bureaucracy, Health services research, Primary care, General practice

## Abstract

**Introduction:**

In care settings across the globe non-clinical staff are involved in filtering patients to the most appropriate source of care. This includes primary care where general practice receptionists are key in facilitating access to individual surgeries and the wider National Health Service. Despite the complexity and significance of their role little is known of how the decision-making behaviors of receptionists impact policy implementation and service delivery. By combining the agent-based implementation theory of street-level bureaucracy with a tri-level analytical framework this work acknowledges the impact of the decisions made by receptionists as street-level bureaucrats and demonstrates the benefits of using the novel framework to provide practical insight of the factors influencing those decisions.

**Methods:**

A secondary analysis of qualitative data gathered from a series of semi-structured interviews conducted with 19 receptionists in the United Kingdom in 2019 was used to populate a tri-level framework: the *micro-level* relates to influences on decision making acting at an individual level, the *meso-level* influences at group and organizational levels, and the *macro–level* influences at a societal or policy level.

**Results:**

At the *micro-level* we determined how receptionists are influenced by the level of rapport developed with patients and would use common sense to interpret urgency. At the *meso-level*, influences included their position at the forefront of premises, the culture of the workplace, and the processes and protocols used by their practice. At the *macro-level*, participants described the impact of limited health service capacity, the lack of mandatory training, and the growth in the use of digital technologies.

**Conclusions:**

Street-level bureaucracy, complemented with a tri-level contextual analysis, is a useful theoretical framework to understand how health workers, such as receptionists, attempt to provide universality without sufficient resource, and could potentially be applied to other kinds of public service workers in this way. This theoretical framework also benefits from being an accessible foundation on which to base practice and policy changes.

## 1 Introduction

In health care systems across the world, non-clinical staff are frequently expected to filter patients towards the most appropriate source of primary care. In the United Kingdom (UK), this primary care consists of dental health, optometry, community pharmacy, and general practice. The position of the latter at the gateway to the other primary care services and the broader National Health Service (NHS) means individual general practice organisations and their staff have a key role to play in the equitable, integrated, and responsive NHS envisioned by policymakers [1,2]. However, primary care in the UK is operating under considerable and growing pressure from an ageing population, exacerbated by a COVID inspired backlog, funding constraints, and a workforce that continues to lose experienced clinicians [3]. This leaves general practice in particular squeezed between the NHS's aspirations of universality and the realities of increasing patient demand and shrinking clinical capacity [4] and has prompted the Royal College of General Practitioners (RCGP) to call for an urgent exploration of the processes that underpin access to primary care [5].

Situated at the organizational periphery of general practice, reception staff have traditionally borne the majority of the responsibility for fielding patient requests for clinical access [6]. This includes prioritizing patients for clinical consultations with practice staff or directing patients to acute care or third-party sources of information and support [7]. The steps involved in patient assessment and service designation performed by receptionists are summarised in Supplementary File 1. In performing this role, they are directly exposed to the tensions between a demand that exceeds capacity, making key decisions on access in complex and time-pressured environments [8,9].

Traditionally, publicly-funded front-line service providers charged with meeting the needs of service users were considered to have negligible influence on policy implementation and service delivery until Lipsky recognized the power of their discretionary decision-making when balancing the preferences and needs of clients and the capacity and goals of their parent organisation all whilst minimising work-related stress [[10], [11], [12]]. This discretion is particularly conspicuous where demand outstrips supply when it is used to help resolve excessive workloads, manage complex cases, and meet often ambiguous performance targets [11,13,14]. As such it is now recognized that the routines front-line service providers establish, and the devices they invent to cope with uncertainty and pressure means they reshape the policies they deliver [13]; the leeway they are granted contributing to the gap between policy as intended and policy as delivered [11,[13], [14], [15]]. Subsequently defined and explored as ‘street-level bureaucracy’, this agent-level implementation theory attempts to explain how workers' individual beliefs, personal characteristics [16], and a range of contextual influences including that of service users, colleagues, and their organization, act upon them as they deliver and potentially re-interpret local and national policy [11,13,15,17,18].

As a concept, street-level bureaucracy has been successfully used to describe the influences on the decision-making behaviours of a range of front-line healthcare providers including nursing staff in Ghana [19] and Scandinavia [20] and GPs in India [21]. The evidence suggests their discretion can impact on the way they process patients and ration access to care [22] though in confronting scarcity and uncertainty in healthcare these decisions are neither costless or frictionless [11,23,24]. Street-level bureaucrats (SLBs) were initially understood as professionals though it is now argued that they are better understood as an “organizational caste” [25] defined not by their training or qualifications but by the characteristics of their role and their relationship to managers, service users, and external parties [26]. We argue that general practice receptionists can be considered SLBs and the work we present here seeks to apply the concept to their role in general practice to better understand the impact of their decision making on the delivery of primary care [14], adding to the theoretical literature on street-level bureaucracy by demonstrating the value of the approach in the context of access to general practice.

To maximise our learning we need to understand that receptionist decision-making is “nested within the context of routines, practice ideologies, rule following, and law” [27]. However, much of the existing research on street-level bureaucracy in health care has focussed on the interaction between individual provider and service user (patient), limiting how effectively we can understand the influences of policy and organisational context [28]. To help understand the nature and scope of the contextual influences that operate on receptionists we follow the recommendations of Gofen et al. (2019) who, in pursuing a more rounded grasp of the decision-making “space” in which SLBs operate, suggest a tri-level analytical approach that not only explores the influence of direct contact with service users (at the *micro-level)* but also the impact of their organization (at the *meso-level)*,and overarching policy (at the *macro-level)* [29].

The data analysed here were sourced from a series of semi-structured interviews conducted with receptionists as part of a larger study of their role in the context of modern primary care [30]. Our findings provide unique insight into the work of receptionists within the resource-constrained environment of UK primary care. Enhanced by the tri-level contextual analysis it has allowed us to place the receptionists’ impact amongst the broader influences acting on primary care delivery and within the context of other SLBs operating in the public sector.

## 2 Methods

### 2.1 Study design and research team

The work we present here used a (secondary) directed content analysis to populate a tri-level framework informed by the agent-focused implementation theory of street-level bureaucracy [11,29,31]. The research team included three medical sociologists and a postgraduate researcher with experience of qualitative research methodologies [32].

### 2.2 Settings, recruitment, and data gathering

The interviews were undertaken in 2019 across five practices recruited from within the West Midlands, an area with an estimated population of nearly 3 million and a broad ethnic and socio-economic mix [33]. Maximum variation sampling [34] was used to ensure practices were recruited with a range of patient list sizes, number of GPs employed (whole-time equivalent) and a broad range of socio-economic environments based on indices of multiple deprivation [35]. The process of recruitment was supported by the Clinical Research Network West Midlands [36] and the number of participants estimated as appropriate for achieving saturation in the initial analysis [37]. Written informed consent was obtained prior to the interviews, which were conducted face-to-face within the participants' workplace at a time that met their preference [38] by [3rd author], a non-clinical, white, male researcher who was previously unknown to the participants. The interviews explored receptionists’ roles, through a topic guide that asked questions about the tasks performed, and the influences of patients, colleagues, and the broader health-system. A summary of the topic guide can be found in Supplementary File 2.

### 2.3 Data management, analysis, and theory development

All interviews were digitally recorded and transcribed verbatim by a professional transcription company and the data managed using NVivo 10 (https://www.qsrinternational.com/).

The original analysis of the data set used a qualitative descriptive approach which allowed the creation of a process map describing the roles of receptionists in triaging patients in general practice [32]. In developing the process map it became clear that the richness and the complexity of the data offered potential for additional insight into the often unseen and unrecognized elements of receptionist decision-making. This motivated the performance of a secondary analysis which, following consultation with the literature describing the actions of similar front-line public sector workers, guided us toward street-level bureaucracy as the most appropriate theoretical lens, specifically drawing on Gofen's tri-level framework to highlight the various levels of contextual influence acting on receptionists [30]. This secondary directed content analysis was undertaken by two authors experienced in qualitative research with a background in medical sociology and health services research: a white male [1st Author] and a white female [4th Author]. The data were reanalysed and deductively allocated to the three levels of influence and the themes and sub-themes within these identified inductively, all were consensually agreed [29]. The scope and characteristics of the three levels are further described and defined in Box 1.

Box 1 **Interpretative framework of levels of influence on SLB decision-making behavior**

**Table:** 

*Domain*	*Scope*	*Level of influence*	*Defined by*
*Micro-*	Social interaction between dyads and within smaller groups including between providers and between care providers and service users. It includes oral and written communication.	Individual	Characteristics of individuals including knowledge, attitudes, gender, age, beliefs, and capabilities.
*Meso-*	The social interplay and networks that occurs between groups, networks and organisations.	Organizational	The formal and informal routines and support systems that exist between workers, co-workers, and their organization, and those that exist between organisations.
*Macro-*	The interaction between societal and organizational structures and policies, and systems.	Policy	National policies and guidance, and the broader model or system of provision within which the organization sits.

Alt-text: Box 1

## 3 Results

### 3.1 Sample characteristics

Receptionists were interviewed from five sites that possessed characteristics broadly representative of the national range of general practices [39]; three were in some of the most deprived areas of England (Practices −02, - 03, and −05), and two in some of the least deprived (Practices −01 and −04). They supported a range of patient list sizes, and numbers of whole time equivalent (wte) GPs. A total of 19 receptionists participated across the five practices with interviews lasting between 25 and 43 min: all participants were white women. Table 1 summarises the characteristics of participating practices and the number of participants interviewed at each.

**Table 1:** Participating practices and their key characteristics.

General Practice	Practice Characteristics				Participants
	List Size	Number of GPs (WTE)^a^	Practice Size	IMD rank and Decile^b^	No. of receptionists interviewed
*P01*	11,114	5	Medium	21,688 (7th)	5
*P02*	8211	4	Small	3360 (2nd)	4
*P03*	21,847	11	Large	6166 (2nd)	4
*P04*	7193	3	Small	31,280 (10th)	3
*P05*	7632	4	Small	9998 (4th)	4

a Whole time equivalent.

b IMD: Decile 1 = most deprived; 10 = least deprived (Ministry Of Housing Communities & Local Government, 2017).

**Fig. 1:**
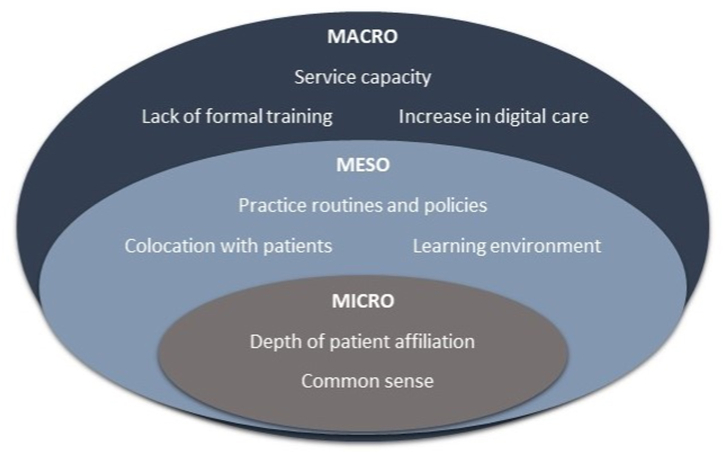
Summary of key influences on GP receptionist discretionary decision making.

A full description of our findings are presented below within the three domains of our tri-level framework alongside exemplar quotes below identified by their participant number and practice code to prevent compromising their confidentiality [40]. A summary of the domains and sub-themes is presented in Fig. 1.

#### 3.1.1 Micro-level influences

This level describes the interactions between receptionist and patients at the micro-level within two key areas; the depth of affiliation they felt with their patients, including the impact of the attitudes and approaches patients adopted trying to secure appointments; and the reliance on what participants described as their “common sense” to inform their decisions.

### 3.2 Depth of patient affiliation

The rapport developed with patients is an important component of receptionist work and at its optimum can support more appropriate care and service utilization [41]. One participant described how the familiarity they developed with certain patients became a genuine emotional connection:

“The regulars that come up, you do get to know them - but there’s patients that don’t really come up and you don’t really get that bond with them - but yeah, it’s nice to see the patients that you do get a bond with when they come in and they say, ‘Morning. You alright?’ It is nice …” *Receptionist 3 (R03) - Practice 01(P01)*

The frequency of the interactions with some patients generated an understanding of a patient's social circumstances and participants described how this could lead them to intervene on a patient's behalf. For example, if they knew a patient was isolated or vulnerable, they might be more predisposed to seek the GP's involvement:

“… if somebody’s lonely and they’ve got no family we do find they call up and they’ll keep you talking for ages, and you’re thinking, ‘I’ve got to go!’, but you’ve got to think of their side as well, they’ve got nobody … If they’ve got an illness and they’re worrying it is nice for them to have somebody to talk to and we’ll always try and like advise them the best things we can, but if they’re ever stuck, we’d just say, ‘Look, we’ll get a doctor to give you a call. Don’t worry we’ll sort it!’ and they’re always grateful.” *(R04/P01)*

Receptionists have traditionally been recognized and treated as autonomous ‘gatekeepers’ by patients and held independently responsible for restricting access [8]. This continuing presumption was described by our participants who described how some patients were inclined to put excessive pressure on them to meet their preferences:

“… there are some really demanding, really, *really* demanding patients and you’ll pick the phone up … and you’ll know their voice and you just think ‘this is gonna be a hard ten minutes!’” *(R04/P01)*

Participants also described how some patients failed to understand that as receptionists they are expected to follow pre-defined practice procedures and routines:

“I think patients think that we don’t want to help, maybe that we’re kind of like a barrier, but that’s not the case, we still have a job to do - just because they’re saying ‘We need this, this, and this’. If we turned round and said that to the doctor, they would be the ones that would be like ‘Oh? Well, you know it doesn’t work like *that*’. So, I think sometimes they forget it is a job role, we have to abide by certain rules and protocols …” *(R02/P02)*

These routines include receptionists asking rudimentary questions about why they needed to see a clinician in the process of allocating care [42]. However, not every patient was happy to discuss potentially sensitive health information with unqualified receptionists:

“We do ask them why they want the call from the doctor [and] there is the occasional person that says, ‘You’re only a receptionist, what do you want to know for?’ *(R04/P02)*

Participants also recognized how some patients would attempt to ensure their expectations for treatment were met by manipulating these conversations using ploys such as exaggerating their symptoms:

“People play the system, they will tell you it’s a lot worse, or they will give you an example of something that’s really wrong but when they see the GP it’s something completely different!” *(R05/P01)*

### 3.3 Common-sense

In UK general practice, receptionists are expected to consult colleagues in difficult cases [43]. However, the criteria as to which incidents required advice appeared vague and we found participants describing how they would use their “common sense” to determine which cases warranted the input of more senior staff:

“Some things I would feel comfortable making a decision on whereas other things my flags might start going up and thinking ‘Right, okay, I cannot take responsibility for saying this’ and ‘If I’m wrong it falls back onto me and something happens then it’s not good’ … I think you have to use your own common sense like ‘Is this something that I can happily make this decision on, or do I question it and take it further?’” *(R02/P02)*

Participants also described how they would use this “common sense” when considering the urgency of an appointment request:

“Sometimes, it’s what we say is ‘common sense’ … that if someone’s coming in - I don’t know, that are on the pill or that they need an injection, or anything like that - then the things that can wait till maybe the next day, then we will put for the next day.” *(R02/P01)*

#### 3.3.1 Meso-level influences

Participants described three key areas of influence on their decision-making at the meso-level. These related to their unique position as a prominent point of first contact; the extent to which their organization possesses a learning environment; and the impact of practice specific routines and protocols.

### 3.4 Colocation with patients

Within the practice organization, receptionists are required to negotiate the often-conflicting demands of their practice colleagues (including administrative and clinical staff) and patients [42]. Their unique position as intermediary is further underpinned by their physical location in the practice where they frequently sit apart from other practice staff, sharing their working environment with patients in reception or waiting areas [44]. Participants described how using the additional insight into individual patients and the organization gained from this position could be used to the advantage of both patients and organization:

“… if the doctors need something you’re there to help them, but at the same time you’re helping the patient to get the right care that they need. So, yeah, you’re helping both of them really, being in the middle, getting to see all kinds of points of view.” *(R04/P01)*

Participants also described how their location in practice premises led to them feeling their work can go unseen by other staff and that the full extent of their role, and the efforts they made in fulfilling its various aspects were not always understood:

“… I do think they appreciate it, but it’s not always, doesn’t always come across and then that can kind of grind you down a little bit you know? I will go above and beyond for people that appreciate me, but I think it’s a bit of a downer when you feel like you have and there’s no … [acknowledgment]” *(R02/P02)*

### 3.5 Learning environment

There are no formal qualifications required to become a receptionist and the current NHS job description for the role of general practice receptionists, describes their need to learn quickly as part of a team [44]. In relation to this our participants described how they would readily seek informal advice from both clinical and non-clinical colleagues:

“If my team leader isn’t in, I know I [can] pick up the phone and ask the manager. If she doesn’t know? I’ll ask a doctor and if they don’t know? I’ll just keep asking *all* the other admin staff until I get to the one with the most experience!” *(R01/P02)*

At another practice, a participant described how access to the advice of colleagues played an important part in alleviating some of the pressure of making potentially critical decisions:

“… if I’ve got a query and I didn’t feel comfortable to ask anybody then I think it’d eat away at me and it’d get me down … I’d be stressed and worried that I’d done something wrong. So, I think it is very important to have a good bond with the people you work with … “ *(R04/P01)*

### 3.6 Practice routines and policies

Each English general practice has the autonomy to design the processes and systems they use to manage the flow of their patients through their organisation, guided by best clinical practice, the local priorities of primary care networks and the expectations of national regulatory bodies [1]. The practice specific processes that result are intended to be flexible enough to reflect the clinical needs and demographic characteristics of individual patients [5]. For example, participants understood the significance of a patient's age when assessing the urgency of their request:

“… if they call up and it’s like, a child with a rash then it’d be to see a doctor ‘cause the nurses don’t deal with children. So, it just depends on what the situation is …. it’s just what I’ve learned over the years of being here …” *(R04/P01)*

Participants also described how their interpretation of symptoms described by a patient would be informed by their understanding of the protocols put in place to help manage certain conditions. For example, one participant described how patients presenting with chest pain would need additional attention:

“We do have all [sorts of] policies … if they ring and they’ve got chest pains and it’s classic for like heart [problems], then it’s an ambulance, but we do have to check a lot because someone will say ‘Oh I’ve got chest pains!’, ‘Ok, what other symptoms have you got?’ and then it’s a chest infection, so we have got to be very careful in saying ‘Oh, call the ambulance!’ …” *(R03/P01)*

Despite their awareness of the various practice protocols and processes, participants described how they would bend these rules where they considered it warranted:

“We ask patients to ring in before five. She phoned me at ten to six and said ‘My leg is swollen! It’s twice the size and its blue!’ … I could have turned round and said to her ‘Well, the duty doctor is not here. Please call back in the morning.’ but I found the doctor and I just said, ‘Can I pop her on the list?’ and he saw her within 20 minutes.” *(R01/P02)*

#### 3.6.1 Macro-level influences

Any individual operating within a publicly funded organization is impacted directly or indirectly by a range of national policies and initiatives [11]. We identified their impact on receptionists’ decision making in three areas; the expectations and requirements for training and qualifications; the effects on service capacity of recent policy; and the early impact of initiatives designed to support the roll-out of digital technologies [1].

### 3.7 Training requirements and provision

While formal qualifications are not required to become a receptionist, and the majority of training is conducted in-house by more experienced administrative staff, courses are available from registered training providers, which cover a number of topics relevant to the responsibilities of reception staff [45], as one participant described:

“We went on a course doing medical terminology and that was really helpful - it was just like a short course but yeah … it made you understand [medical] words a little bit!” *(R02/P01)*

Because training is not mandatory and without dedicated time or resource, uptake of these additional courses varied. For example, one participant enrolled on a course to support safe repeat prescribing were expected to complete the course independently and outside of work hours:

“… for the Prescriptions Medicine Co-ordinator [qualification], we both passed at that, we were the first practices in the area to actually get that done … So, yeah we have had to take on quite a lot of education and do it in our own time to get this qualification …” *(R01/P02)*

### 3.8 Service capacity

The increasing demands placed on general practice in the UK have been compounded in the last decade by real-term cuts to funding and the lack of a long-term plan for the training and recruitment of the primary care workforce [46,47]. Our participants described the lived experience of these structural limitations on their day-to-day work:

“We’ve got a bell now that we press on reception, so that it’ll let the people out the back know that you’re really busy and there’s a massive queue. So, if there’s only two of you there and you’re answering the phones, helping the person out the back … and then it is just bonkers sometimes. Sometimes, I go out of here and I think ‘Oh God, I don’t, I don’t know what I’ve done today … cos I, I can’t, I can’t think!” *(R04/P02)*

Demand regularly outstrips supply of appointments and where once receptionists would suggest calling back the next day a recent mandate by the UK government has dictated that receptionists must direct patients to an alternative source of care [48]. In the meantime this consistent pressure on primary care services has led to general practices adopting various measures in attempts to ensure more serious cases can be prioritised. One common work-around is keeping some appointment slots free so patients with more urgent needs can be seen on the same day. However, requests for these appointments regularly outstripped the number available:

“Roughly about five or six appointments aren’t released until two thirty in the afternoon and they are called ‘emergencies’ but it’s a first come first served basis. Sometimes we could use twenty of them if we had them.” *(R01/P04)*

### 3.9 Technology-enabled access to care

The NHS has taken a strategic decision to increase the use of digital technologies to help cope with the increase in demand for primary care [1]. These include the facility for patients to order repeat medications, view test results and book routine appointments [49]. One participant recognized how patients were increasingly using these options as an alternative to long waits on busy telephone lines attempting to reach reception staff:

“A lot more people are starting to do prescriptions online and booking appointments, it is starting to filter in [to] patients. ‘Cos they don’t want to be sat on the phone at 8 o’clock on a morning, so you offer them the online access and a lot of them do start going to that.” *(R01/P04)*

## 4 Discussion

### 4.1 Theoretical contribution

Receptionists are traditionally understood by policymakers, senior decision makers, and patients as performing a primarily administrative role requiring no previous training or qualifications and of little consequence to how the NHS delivers care. Our study elucidates the key role they play in how successfully general practice facilitates access to care particularly when demand outstrips supply. The perception that only professionals can act as SLBs is no longer the orthodoxy [25] and our study has provided further evidence of how the work of those without professional qualifications can still be usefully interpreted as street level bureaucracy. A second dimension to our theoretical contribution is our focus on context, vital, given the constraints of resource and growing patient demand which general practice must accommodate. To operationalize this in our analysis, we drew on Gofen et al.'s (2019) tri-level framework, which helped us unpick the multiple contextual influences that receptionists are subject to: at the *micro-level* their decisions on access are affected by their interplay with patients and the judicious use of common-sense; and how the space in which receptionists can make life-changing decisions is shaped at the *meso-level* by the structure and culture of the practice organisation in which they work, and at the *macro-level* by the impact of several overarching health policies.

### 4.2 Empirical findings

#### 4.2.1 Micro level influences

Previous work has recognized how SLBs categorise service users based on objective parameters defined by their organisation [50]. Similarly the receptionists we spoke to described how they would use formally described variables based on best medical practice such as patient age or medical history to determine eligibility [51]. It is also worth noting that our participants made decisions informed by ‘common sense’ which though considered an objective judgement of facts [52] can be more precisely understood as an assessment based on tacit knowledge informed by life experience and, therefore, subject to wrongful assumption or bias [53,54]. Our participants also alluded to the influence of more informal assessments of patients based on a prior understanding of the individual and their social characteristics, for example where they were considered vulnerable or lonely.

Street-level bureaucrats (SLBs) in other areas of public service have also described using unofficial categories that lack formal definition [55], relying on perceptions of eligibility and urgency informed by prior experience of individual service-users [12,13,15,56]. This includes perceptions of social characteristics such as class and ethnicity [20,57], where SLBs have demonstrated greater leniency in supporting requests of those with whom they share social demographic characteristics [58].

General practice receptionists are predominantly female, white, and middle aged [45] and it is feasible that the predominance of women may deter men, who are often more reluctant users of health services [59]. The lack of ethnic representation amongst reception staff could also prove a barrier to patients from minority ethnic groups accessing care due to a lack of culturally specific understanding [60,61]. Though the influence of receptionist demographics on subsequent decision-making has yet to be explicitly explored there is evidence elsewhere in the NHS that increasing the diversity of administrative staff has improved access to underserved populations [62].

Participants described how some patients were notoriously ‘difficult’ when seeking access, placing receptionists under excessive pressure to meet their preferences. This perspective may reflect a societal perception that receptionists can obstruct access and explain why receptionists remain amongst the most complained about members of the general practice team [63] and increasingly subject to open abuse [64]. The patient-led pressure on receptionists has risen in recent years, exacerbated by difficulties of gaining access to primary care during the COVID pandemic [5]. Though attributable in part to the shortfall in GP availability, other factors are likely at play; for example, patients have been encouraged by the NHS to consider themselves consumers, a concept introduced in expectation of generating greater individual satisfaction but has instead led to increased confrontation with care providers [65]. Another explanatory factor may be the narrative perpetuated by some politicians and mainstream media outlets that blames individual general practice staff for the shortfall in available appointments [66]. It might be reasonably assumed that against this backdrop receptionists might feel intimidated into meeting the preferences of more confrontational patients, however this is not necessarily the case. Those we spoke to intimated that they are more likely to accommodate those that understand and follow the system as intended, a phenomenon observed amongst SLBs in other public sector settings [15].

#### 4.2.2 Meso-level influences

Participants described how their colocation with patients enabled informal data gathering, and added valuable context to their communications with clinicians, though it also led to their feeling unseen and undervalued, as reported previously amongst general practice receptionists [43]. There is evidence that physically isolated SLBs and those encountering a similar lack of acknowledgement from senior staff are less likely to follow the policies and routines of their organisation, relying on their own discretion when rationing access [67,68].

Participants described the importance of the advice of colleagues and senior staff across an increasingly multi-disciplinary practice team [69] and previous work has described how the relationship between SLBs and their supervisors helps shape the areas of the service where they use their discretion [70] including when to consult senior staff [71]. Ultimately developing the level of rule-compliance of more experienced colleagues [72]. For example, practice specific protocols and processes appeared to be understood by our participants, but they used their discretion in how closely they were followed with the tacit agreement of senior staff. These protocols are not always well designed or defined [9] and GPs have also exhibited similar discretion in their pursuance [14].

Participants also described their role as “gatekeepers” to the broader National Health Service, determining whether emergency care was required in our example from patient descriptions of chest pain. This element of their role is now being expanded, reformed, and renamed as “care navigators” or “coordinators” [73] and National Health Service England have introduced formal navigation training which includes recognising serious cases and signposting people to sources of advocacy and support, including from local community groups [74]. Similar navigator roles, and associated training, are being created in other healthcare systems internationally particularly where lay staff are required to perform triage roles [75].

#### 4.2.3 Macro-level influences

Although street-level bureaucracy was first introduced as a way of understanding how the actions of individuals influenced policy implementation it also acknowledges that policy directly and indirectly shapes the actions of SLBs [13,29]. In the NHS, the lack of a coherent workforce strategy, funding sufficient to meet inflationary pressures, and the shifting demography of the population, has led to unprecedented pressure being placed on the entire health service but particularly primary care [47]. Our participants described the daily pressures meeting patient preferences for access though it is recognized that not every patient needs or requires same-day appointments with their favoured GP, though maintaining continuity of care for those most likely to benefit is reliant on the capacity of the surrounding health economy to address urgent cases [76].

Receptionists are often implicitly expected to manage excessive demand, though this has yet to be acknowledged by policymakers and senior staff [5]. This issue is not confined to the UK, globally lower and middle income countries are faced with a similar shortage of clinical staff with those providing access charged with similar levels of responsibility [77]. In other public sector organisations, the discretionary decision-making of SLBs is more consistently applied to reduce acute issues of supply and demand [18,78]. However this reliance is not without risk and there is evidence that SLBs in English social care have made inappropriate decisions whilst attempting to address complex cases whilst experiencing high demand [79].

Participants reported patients’ increasing use of the digital tools made available by the NHS that to facilitate remote access, including algorithm driven triage and online booking systems [1,49]. Improving the consistency of access through digital technologies is driving reform in other public sector services such as social work [80] though in health or social care these digital tools run the risk of favouring wealthier, better educated, and more digitally literate individuals [81]. They also risk compromising the benefits of the contextual understanding of individual patients by receptionists [41,81]. Associated with the shift to remote access is “NHS 111” a triage only service designed to provide advice and signposting to appropriate services for people with urgent health-care problems [82], however it remains under utilised by the most frequent GP attenders namely older or less well-educated patients [83] with evidence of it causing duplication of effort for patients and providers [76,82].

In healthcare systems in Australia and elsewhere there have been recent moves to ‘professionalise’ receptionists by increasing training and qualifications [84,85]. Theoretically at least, professionals are deemed more capable of balancing the needs of individual clients with wider organizational and economic contexts [86] and evidence is emerging that greater professionalisation has improved discretionary behaviours in SLBs working in welfare and social support [70,87]. Despite the growing recognition in the UK that the *ad hoc* training regimes our participants described are being improved by better support for signposting, the formal professionalisation of the role in general practice is yet to be seriously discussed [73].

#### 4.2.4 Strengths and limitations

This is the first time that the work of general practice receptionists in the UK (and elsewhere as far as we are aware) has been explored through the implementation theory of street-level bureaucracy. The secondary analysis followed best practice [31] supported by the largest qualitative exploration of receptionists yet conducted in the UK, a member of the primary care team that researchers frequently overlook. The sample incorporated participants drawn from a range of practice organisations serving a diverse range of patient bodies [39]. Although the sample size was relatively small we hold that it remains a viable and valid data set as supported by consensus theory, i.e., that they are ‘experts’ sharing knowledge and common values and accounting for the absence of negative cases [88,89]. Given this was a secondary analysis, we did not have data directly on how receptionists experienced and reflected on their SLB role, which would be an area for future research. This includes their experiences of rationing care alongside the perspectives of patients and other members of the practice team. This would be aided by including observational methodologies and a sampling frame to draw out maximum variation in the demographic characteristics of the receptionists and the populations they serve to explore any variation or disagreement.

The data were collected before the onset of COVID, and the changes put in place as a result of the pandemic to support telecare such as total triage [90], means that patients now more frequently rely on digital tools to access care [91]. There is also more widely established multi-disciplinarity in the primary care team [92]. Despite these changes it is expected that receptionists will continue to be relied upon to perform many aspects of their traditional role [93].

The rigour of our work was upheld by employing a number of recommended strategies; clear and accurate records of the progress of the work were shared across the team [94], we were open about the experience and prior knowledge of the interviewer [95], and have used rich and verbatim descriptions of participants’ comments [96]. In addition, the initial stages of the data analysis were undertaken by two researchers working independently [94].

The tri-level analytical framework proved an effective means of exploring the range of often unseen or unrecognized influences on receptionist decision-making behaviours beyond the individual negotiation with patients. In doing so we could draw on the lessons learned from SLBs in other public sector settings within the broader *meso-* and *macro-*levels of influence and, reflecting the success of similar multi-level frameworks in healthcare research, provide a structure that promotes a shared understanding for intermediaries and practitioners, [97]. Therefore, it has provided an important first-step towards promoting a more thorough understanding amongst policy-makers, commissioners and senior decision-makers of the influences that act upon receptionists and other SLBs that ration access.

## 5 Conclusions/implications for practice or policy

The role of receptionists in UK general practice has always been ambiguous, oscillating between assumptions of process driven administrators and autonomous, discretionary decision-makers. Their role is increasingly conditioned by NHS attempts to provide person-centered universality with shrinking resources and using the lens of street level bureaucrats has provided a more nuanced understanding of their role in facilitating access to primary care and the wider health service as well as the range of influences on their decision-making behaviour that extends beyond the interaction with the patient to include the organizational and the broader environmental influences of policy and health system.

## Ethics statement

Ethical approval was granted by the NHS Health Research Authority (117/WM/0203) and the University of Birmingham's Science Technology Engineering and Medical ethical review committee (ERN_15-1175).

## Data availability statement

The data associated with the study has not been deposited into a publicly available repository. This is because data that has been used is confidential.

## Funding Statement

This work was supported by The Health Foundation grant number 7452. They played no role in the design of the study, the collection, analysis or interpretation of the data, and the content or editing of this manuscript.

## CRediT authorship contribution statement

**Ian Litchfield:** Conceptualization, Formal analysis, Funding acquisition, Methodology, Resources, Supervision, Writing – original draft. **Nicola Gale:** Conceptualization, Funding acquisition, Investigation, Methodology, Supervision, Writing – review & editing. **Michael Burrows:** Data curation, Formal analysis, Investigation, Methodology, Project administration, Writing – review & editing. **Sheila Greenfield:** Conceptualization, Formal analysis, Methodology, Supervision, Writing – review & editing.

## Declaration of competing interest

The authors declare that they have no known competing financial interests or personal relationships that could have appeared to influence the work reported in this paper.

## Figures and Tables

**figs1:**