# Supplementary material for: “You're only a receptionist, what do you want to know for?”: Street-level bureaucracy on the front line of primary care in the United Kingdom
Source: Heliyon. 2023 Nov 13;9(11):e21298. doi: 10.1016/j.heliyon.2023.e21298 (PMC10694055; doi:10.1016/j.heliyon.2023.e21298)
Supplement: Multimedia component 1 [file mmc1.pdf]

Supplementary File 2: Summary of topic guide for semi-structured interviews with receptionists

- What roles do you perform within your surgery?
    - What are the processes involved in undertaking those tasks (e.g. timing, procedures)?
    - Do you consider any of these roles as clinically orientated?
- What do you consider are the key issues or difficulties associated with fulfilling these roles?
    - Resource, training, patient expectations, others
- What support, training or guidance has been offered to you to help perform these roles
    - Via practice staff or external training providers
- How do you think your role is perceived by colleagues and senior staff?
    - Is it different between clinical and non-clinical staff?
- What do you think are the future challenges facing the GP receptionist?
    - Funding, digital technologies, patient demands
